# Glucocorticoid Minimization in Anti-Neutrophil Cytoplasmic Antibody-Associated Vasculitis: An International Survey of Clinicians

**DOI:** 10.1016/j.xkme.2024.100858

**Published:** 2024-06-14

**Authors:** David Massicotte-Azarniouch, Mark Canney, Priscilla Karnabi, Peter A. Merkel, Rachel B. Jones, Ruth J. Pepper, Alan D. Salama, Vimal K. Derebail, Nataliya Milman, Mats Junek, Christian Pagnoux, David R.W. Jayne, Michael Walsh

**Affiliations:** 1 Division of Nephrology, The Ottawa Hospital, University of Ottawa and Ottawa Hospital Research Institute, Ottawa, ON, Canada; 2 Clinical Epidemiology Program, the Ottawa Hospital Research Institute, Ottawa, ON, Canada; 3 Division of Rheumatology, Department of Medicine and Division of Epidemiology, Department of Biostatistics, Epidemiology, and Informatics, University of Pennsylvania, Philadelphia, PA; 4 Renal Medicine, Addenbrooke’s Hospital, Cambridge, United Kingdom; 5 University College London Department of Renal Medicine, Royal Free Hospital, London, United Kingdom; 6 Division of Nephrology and Hypertension, Department of Medicine, UNC Kidney Center, University of North Carolina at Chapel Hill, Chapel Hill, NC; 7 Division of Rheumatology, The Ottawa Hospital, University of Ottawa and Ottawa Hospital Research Institute, Ottawa, ON, Canada; 8 Division of Rheumatology, Department of Medicine, McMaster University, Hamilton, ON, Canada; 9 Division of Rheumatology, Vasculitis Clinic, Mount Sinai Hospital, University Health Network, Toronto, ON, Canada; 10 Department of Medicine, University of Cambridge, Cambridge, United Kingdom; 11 Division of Nephrology, Department of Medicine, McMaster University, Hamilton, ON, Canada

**Keywords:** Anti-neutrophil cytoplasmic antibody-associated vasculitis, cyclophosphamide, glucocorticoid, rituximab, survey

## Abstract

**Rationale & Objective:**

Research in anti-neutrophil cytoplasmic antibody-associated vasculitis (AAV) has focused on reducing treatment toxicities, notably through reduction of exposure to glucocorticoids. Glucocorticoid-sparing therapies such as avacopan are not widely available in many countries, and patients are exposed to high glucocorticoid doses. There is little data concerning what clinicians should accept as the lowest glucocorticoid dosing that can be used in induction therapy for AAV.

**Study Design:**

International, online survey.

**Setting & Participants:**

Clinicians in various countries with experience in managing vasculitis.

**Exposure and Outcomes:**

Survey questions to gauge interest and preferences in studying an induction of remission regimen for severe AAV using only 2 or 4 weeks of glucocorticoids without avacopan. Data collected included general opinions about standard of care for induction agents, glucocorticoids, and avacopan. Respondents were presented with 3 candidate trial designs, 2 of which proposed a combination of cyclophosphamide and rituximab induction.

**Analytical Approach:**

Using a 10-point Likert scale, respondents ranked each candidate trial on its usefulness in demonstrating whether a minimal glucocorticoid regimen would be safe and effective and their willingness to randomize into the trial.

**Results:**

There were 210 respondents to the survey. The candidate trials were rated moderate-to-high for usefulness to demonstrate safety and efficacy (scores 6-7/10), and moderate (scores 5-6/10) for willingness to randomize. Four-week glucocorticoid duration was preferred to 2 weeks, and combination cyclophosphamide-rituximab with 4-week glucocorticoids was the most preferred design. Forty-two percent of respondents felt avacopan had to be incorporated into a minimal GC trial design to want to recruit patients.

**Limitations:**

Representativeness of survey sample and generalizability of findings.

**Conclusions:**

Combination cyclophosphamide-rituximab may be the ideal way of studying minimal glucocorticoid use in severe AAV. Given its increasing uptake, incorporating avacopan into a potential trial design is important.

## Plain Language Summary

Research in anti-neutrophil cytoplasmic antibody-associated vasculitis (AAV) has focused on using less glucocorticoids to limit side effects. New drugs that drastically limit glucocorticoid use are not available in many countries. Studies are needed to find other ways of reducing glucocorticoid exposure to treat AAV, but it is unclear how best to achieve this. We administered a survey to doctors with experience in treating AAV and had them grade different combinations of widely available treatments with 2 or 4 weeks of glucocorticoids. We found that a combination of 2 doses cyclophosphamide with 2 doses rituximab and 4 weeks of glucocorticoids was the preferred treatment. The results will guide the development of a trial studying minimal use of glucocorticoids for the treatment of AAV.

Anti-neutrophil cytoplasmic antibody-associated vasculitis (AAV) is a systemic autoimmune condition that can lead to organ failure and even be life-threatening. A mainstay of therapy for AAV has long been glucocorticoids (GCs), which offer rapid and often substantial benefits for disease control and symptom resolution. However, prolonged use of GCs at high doses leads to significant side effects that both patients and clinicians fear.^1^ Much research has gone into decreasing the amounts of GCs required to effectively treat AAV while maintaining adequate disease control.

The Plasma Exchange and Glucocorticoid Dosing in the Treatment of Anti-Neutrophil Cytoplasm Antibody Associated Vasculitis (PEXIVAS) and Low-dose Glucocorticoid Vasculitis Induction Study (LoVAS) trials demonstrated that, compared to the previous standard of care GC regimen, marked reduction in the amount of GCs used to induce remission of moderate to severe AAV was possible, significantly reducing therapy-related adverse events while maintaining efficacy.^2^^,^^3^ The ADVOCATE trial showed that the C5a receptor antagonist avacopan could allow marked reduction in GCs with removal within 4 weeks of the start of induction therapy.^4^ However, avacopan has limited availability throughout the world and is costly, and its role in the most severe forms of AAV is unclear. A non-controlled prospective cohort study suggested that GC minimization with only 1-2 weeks of GCs is possible with the combination of cyclophosphamide-rituximab at the time of induction therapy.^5^ Such a GC minimization strategy would provide patients with AAV a means to treat their disease with minimal exposure to GCs and by using the more widely available induction agents cyclophosphamide and rituximab. Interest in such a regimen within the vasculitis community is unclear, nor is the level of GC minimization that clinicians would accept given the current landscape of novel GC-sparing therapies such as avacopan.

We developed a survey for clinicians experienced in treating vasculitis to assess the degree of GC minimization that would be considered acceptable and feasible for induction therapy for severe AAV, without avacopan, in a randomized controlled trial (RCT). Understanding that AAV is a rare disease and that an RCT would require collaboration from multiple centers to achieve sufficient recruitment, the survey and its development process could guide the design of the optimal and most suitable trial to examine this question. Considering a setting of induction therapy for severe AAV, our survey had the following objectives: (1) gather opinions on the current role of GCs, avacopan, and combination induction with cyclophosphamide and rituximab; and (2) gauge interest in participating in a GC minimization trial for induction therapy of severe AAV.

## Methods

### Survey Development

Institutional research and ethics board approval for conducting this research was obtained from the Ottawa Hospital Research Institute in Ottawa, Ontario, Canada. The first iteration of the survey, the pilot survey, was designed by 3 nephrologists (DM-A, MC, and MW). This was inspired by the treatment regimen used in the non-controlled study by Pepper et al.^5^ This pilot survey was presented to a group of collaborators with experience in managing AAV and interest in studying GC minimization (PAM, RBJ, RJP, ADS, VKD, NM, MJ, CP, and DRWJ); it underwent clinical sensibility testing to assess the survey’s validity, clarity, utility, and redundancy (see Item S1 for clinical sensibility testing results).^6^^,^^7^ With the results of clinical sensibility testing, and after gathering comments from our collaborators, adjustments were made to the survey questions. The pilot survey also allowed us to determine the composition of candidate trial designs most suited for presentation to a broader audience of clinicians. A candidate trial with rituximab but no cyclophosphamide or avacopan, along with minimal GCs, was withdrawn as an option after consensus from our collaborators that it would not be appropriate in the setting of severe AAV. Before finalization of the survey, it was reviewed once more by our group of collaborators. Patient input was not sought for the survey development as we chose to focus on the clinician perception aspect of minimal GCs for induction therapy of severe AAV, as a first step in an eventual protocol development process.

### Survey Content

**Figure 1:**
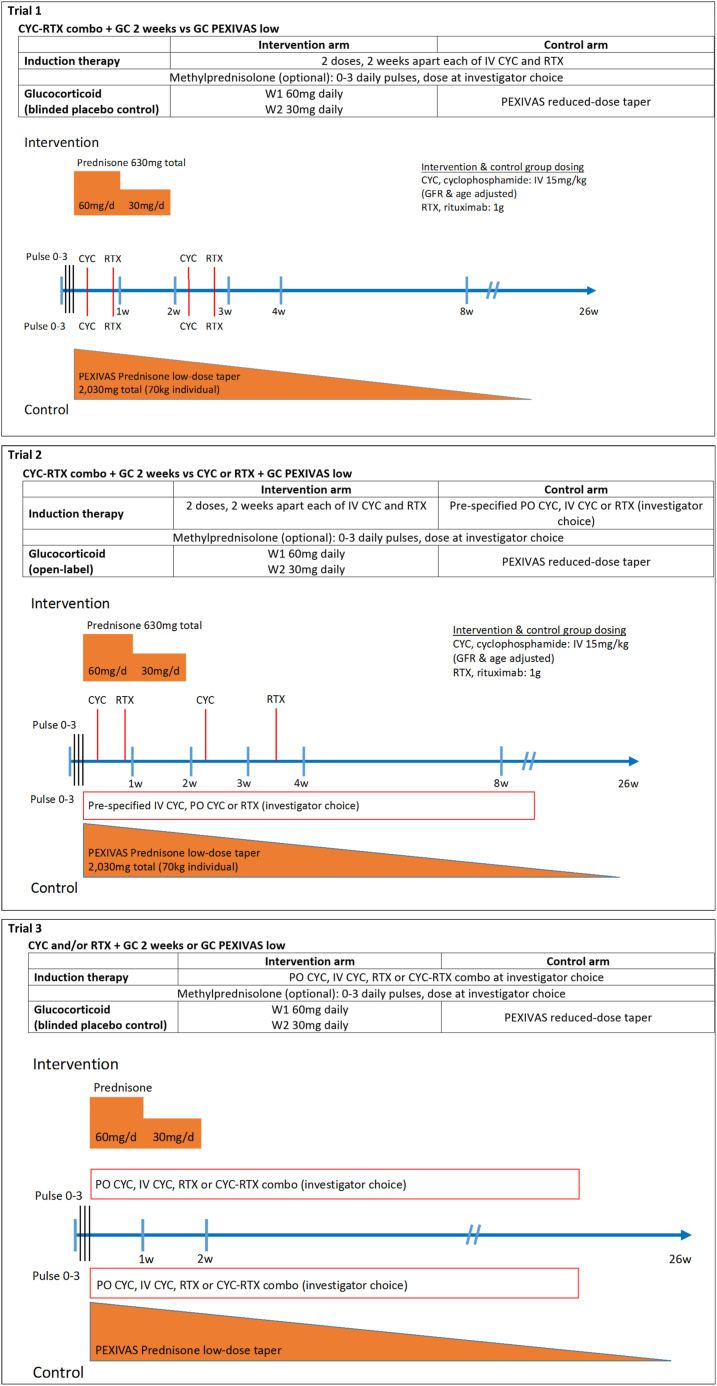
Summary schemas of candidate trial designs for survey. Abbreviations: CYC, cyclophosphamide; GC, glucocorticoid; GFR, glomerular filtration rate; IV, intravenous; RTX, rituximab.

The final survey consisted of questions on respondent demographics and perceptions on the use of current induction and GC-sparing therapies. The survey then presented 3 candidate trial designs. Each candidate trial had an intervention group consisting of 2 weeks of GCs compared to a PEXIVAS reduced-dose GC taper (see Fig 1 for candidate trial summaries). Trial designs differed in the method of use of cyclophosphamide and rituximab in the intervention and control arms. For each candidate trial, respondents were asked the same 4 questions: (1) rate the design on its ability to answer the question of whether minimal GC use in severe AAV is safe and effective; (2) rate their willingness to randomize patients into such a trial; (3) how a 4-week GC taper (compared to 2 weeks) in the intervention arm would affect their willingness to randomize into the trial (the decision option of 4 weeks was made after comments from our collaborators who tested the pilot survey); and (4) an open question where respondents had the option to provide comments about the trial design. Trials were rated using a 10-point Likert scale (1 being extremely unlikely and 10 being extremely likely). The complete survey can be found in Item S2.

The survey was constructed using Survey Monkey, and a link to the survey was in the invitation email message. The survey only allowed one response from a single IP address. The 3-candidate trial designs were presented in a random order to each respondent. A script accompanied the email stating the purpose of the survey and research. Informed consent was implied if a respondent chose to complete the survey. The survey along with the email script were in English.

### Survey Delivery

The survey target population was clinicians from various countries who manage patients with AAV (specified in the email script and introductory paragraph to the survey). Dissemination was facilitated by personal contacts with clinicians and vasculitis societies. The following societies were contacted, and the survey was sent through email lists to their members: the Canadian network for research on vasculitis, the Vasculitis Clinical Research Consortium, the UK and Ireland Vasculitis Rare Disease Group, the European Vasculitis Society, the Australia and New Zealand Vasculitis Society, and the Groupe Français d’Études en Vascularites. We also sent the survey to vasculitis clinicians in Japan, Brazil, and India through individual connections, who then disseminated the survey to clinicians within their country who manage vasculitis.

### Statistical Analysis

Summary data are reported using counts with percentages for categorical measurements and medians with interquartile ranges (IQR) for continuous measurements. On review of the preliminary results, we stratified the results of our survey by medical subspecialty (nephrology vs rheumatology/general internal medicine/other) and geographic location (Canada/United States vs Ireland/United Kingdom vs Europe [excluding Ireland and the UK] vs Asia/Oceania/South America) because we suspected there could differences in opinions on GC minimization and trial design interest based on these factors; manifestations of AAV can differ in kidney-predominant disease vs non-kidney involvement, and variations in treatment patterns may exist due to local practice habits. We did not perform any statistical testing for significance because the survey results were to serve a descriptive purpose and the subgroup selection was done post-hoc. Statistical analyses were performed using Microsoft Excel.

## Results

### Baseline Demographics

There were 210 respondents to our survey. Because respondents may have received invitations from multiple sources, we could not calculate the response rate. The vast majority (82%) practiced in an academic setting; 41% stated Nephrology was their medical specialty, 39% Rheumatology, 12% General Internal Medicine, and 9% other. Respondents reported experience in managing patients with AAV based on the number of patients followed and the number of times induction therapy was initiated within the prior year (Table 1).

**Table 1:** Demographic Characteristics of Survey Respondents

Characteristics	Count (%) N = 210
Country of practice	
France	37 (17.6%)
Japan	33 (15.7%)
United Kingdom	27 (12.9%)
United States	27 (12.9%)
Canada	18 (8.6%)
Australia	13 (6.2%)
Brazil	7 (3.3%)
Other^a^	48 (22.9%)
Location of clinical practice	
Academic	172 (81.9%)
Non-academic	38 (18.1%)
Year started practice (median, IQR)	2008 (2000-2015)
Medical specialty	
Nephrology	85 (40.5%)
Rheumatology	81 (38.8%)
General internal medicine^b^	26 (12.4%)
Other	18 (8.6%)
Number of times you have started induction therapy for AAV in last year (median, IQR)	8 (4-13.25)
Number of patients with AAV you see during follow-up in a year (median, IQR)	25 (10-60)
Number of AAV trials you’ve been involved in (median, IQR)	1 (0-3)

Abbreviations: AAV, anti-neutrophil cytoplasmic antibody-associated vasculitis; IQR, interquartile range.

a Includes the following countries: New Zealand (2), Ireland (2), India (2), Austria (1), Czech Republic (2), Denmark (1), Germany (4), Greece (2), Italy (4), Lithuania (1), Netherlands (5), Poland (1), Portugal (2), Slovenia (2), Spain (1), Sweden (2), Iceland (1), Norway (3), Switzerland (5), Turkey (2), Ukraine (1), Russia (1), Peru (1).

b 20/26 respondents were from France, where many vasculitis clinicians are internists.

### Current Standard of Care Induction Agents

Eighty-eight percent of respondents agreed that the PEXIVAS reduced-dose GC taper was the standard of care for use of GCs in severe AAV, with similar levels of agreement seen between nephrologists and other specialties, as well as by geographic location. Most respondents did not feel access to cyclophosphamide nor rituximab would be limiting factors for recruitment into a trial (Table 2).

**Table 2:** Opinion on Current Standard of Care Agents for Induction of Remission in ANCA-associated Vasculitis

	Completely Agree	Somewhat Agree	Unsure	Somewhat Disagree	Completely Disagree
A reduced-dose GC taper similar to what was used in PEXIVAS is the current standard of care for GC use in patients with severe AAV; n (%)					
Total	80 (40.8%)	93 (47.5%)	11 (5.6%)	5 (2.6%)	7 (3.6%)
By subspecialty					
Nephrology	43 (51.2%)	32 (38.1%)	3 (3.6%)	2 (2.4%)	4 (4.8%)
Rheumatology/GIM/Other	37 (33.0%)	61 (54.5%)	8 (7.1%)	3 (2.7%)	3 (2.7%)
By geographic location					
Canada/United States	20 (48.8%)	17 (41.5%)	1 (2.4%)	2 (4.9%)	1 (2.4%)
Ireland/United Kingdom	18 (62.1%)	8 (27.6%)	1 (3.5%)	1 (3.5%)	1 (3.5%)
Europe^a^	22 (32.4%)	37 (54.4%)	6 (8.8%)	1 (1.5%)	2 (2.9%)
Asia/Oceania/South America^b^	20 (34.5%)	31 (53.5%)	3 (5.2%)	1 (1.7%)	3 (5.2%)
If IV cyclophosphamide had to be used in the intervention and/or control arms, but were not provided by the trial, I would not be able to recruit patients into a GC minimization trial; n (%)					
Total	23 (11.7%)	21 (10.7%)	10 (5.1%)	33 (16.8%)	109 (55.6%)
By subspecialty					
Nephrology	10 (11.9%)	9 (10.7%)	1 (1.2%)	11 (13.1%)	53 (63.1%)
Rheumatology/GIM/Other	13 (11.6%)	12 (10.7%)	9 (8.0%)	22 (19.6%)	56 (50.0%)
By geographic location					
Canada/United States	2 (4.9%)	2 (4.9%)	4 (9.8%)	13 (31.7%)	20 (48.8%)
Ireland/United Kingdom	1 (3.5%)	1 (3.5%)	0 (0)	3 (10.3%)	24 (82.8%)
Europe^a^	9 (13.2%)	6 (8.8%)	3 (4.4%)	8 (11.8%)	42 (61.8%)
Asia/Oceania/South America^b^	11 (19.0%)	12 (20.7%)	3 (5.2%)	9 (15.5%)	23 (39.7%)
If rituximab had to be used in the intervention and/or control arms, but were not provided by the trial, I would not be able to recruit patients into a GC minimization trial; n (%)					
Total	30 (15.5%)	26 (13.4%)	14 (7.22%)	36 (18.6%)	88 (45.4%)
By subspecialty					
Nephrology	13 (15.85%)	10 (12.2%)	4 (4.88%)	12 (14.63%)	43 (52.44%)
Rheumatology/GIM/Other	17 (15.18%)	16 (14.29%)	10 (8.93%)	24 (21.43%)	45 (40.18%)
By geographic location					
Canada/United States	4 (9.76%)	8 (19.51%)	1 (2.44%)	13 (31.71%)	15 (36.59%)
Ireland/United Kingdom	2 (7.69%)	2 (7.69%)	1 (3.85%)	4 (15.38%)	19 (67.86%)
Europe^a^	9 (13.04%)	6 (8.70%)	7 (10.14%)	9 (13.04%)	36 (53.73%)
Asia/Oceania/South America^b^	15 (25.86%)	10 (17.24%)	5 (8.62%)	10 (17.24%)	18 (31.03%)

Abbreviations: AAV, anti-neutrophil cytoplasmic antibody-associated vasculitis; ANCA, anti-neutrophil cytoplasmic antibody; GC, glucocorticoid; GIM, general internal medicine; IV, intravenous; PEXIVAS, Plasma Exchange and Glucocorticoid Dosing in the Treatment of Anti-Neutrophil Cytoplasm Antibody Associated Vasculitis.

a Includes all countries in Europe (except Ireland and the United Kingdom) as well as Turkey and Russia.

b Includes 33 respondents from Japan, 13 from Australia, 7 from Brazil, 2 from New Zealand, 2 from India and 1 from Peru.

### Interest in GC Minimization

In conjunction with questions concerning GC minimization, 82 (42%) respondents stated that avacopan must be permitted in a GC minimization trial design to want to recruit. This percentage was similar between nephrologists and other specialties but was less frequent for respondents from Europe (29%). Sixty percent of respondents felt 2 weeks of GC, without avacopan, was too little GC and would impair patient recruitment. This sentiment was less frequent among nephrologists (52%) compared to other specialties (66%) and respondents from Ireland/United Kingdom (52%). Of the 60% who felt 2 weeks was too little, the median number of weeks felt to make a trial acceptable was 6; this was 4 weeks for nephrologists and 8 weeks for other specialties (Table 3).

**Table 3:** Interest in Glucocorticoid Minimization for the Treatment of ANCA-associated Vasculitis

	Yes	No
Do you feel that the use of avacopan must be permitted in any AAV GC minimization trial design for you to want to recruit a patient into such a trial?; n (%)		
Total	82 (41.8%)	114 (58.2%)
By subspecialty		
Nephrology	35 (41.7%)	49 (58.3%)
Rheumatology/GIM/Other	47 (42.0%)	65 (58.0%)
By geographic location		
Canada/United States	18 (43.9%)	23 (56.1%)
Ireland/United Kingdom	11 (37.9%)	18 (62.1%)
Europe^a^	20 (29.4%)	48 (70.6%)
Asia/Oceania/South America^b^	33 (56.9%)	25 (43.1%)
Two weeks of GC for induction therapy in severe AAV, without using avacopan and irrespective of the amount of cyclophosphamide and/or rituximab used, is too little GC for me to want to recruit patients into a trial; n (%)		
Total	119 (60.4%)	78 (39.6%)
By subspecialty		
Nephrology	44 (52.4%)	40 (47.6%)
Rheumatology/GIM/Other	74 (66.1%)	38 (33.9%)
By geographic location		
Canada/United States	27 (65.9%)	14 (34.2%)
Ireland/United Kingdom	15 (51.7%)	14 (48.3%)
Europe^a^	39 (57.4%)	29 (42.7%)
Asia/Oceania/South America^b^	37 (63.8%)	21 (36.2%)
What would be the minimal number of weeks of GC that would make a trial acceptable to you?^c^; median (IQR)		
Total	6 (4-12)	
By subspecialty		
Nephrology	4 (4-8)	
Rheumatology/GIM/Other	8 (4-12)	
By geographic location		
Canada/United States	4 (4-8)	
Ireland/United Kingdom	6 (4-8.5)	
Europe^a^	8 (4-12)	
Asia/Oceania/South America^b^	4 (4-12)	

Abbreviations: AAV, anti-neutrophil cytoplasmic antibody-associated vasculitis; ANCA, anti-neutrophil cytoplasmic antibody; GC, glucocorticoid; GIM, general internal medicine.

a Includes all countries in Europe (except Ireland and the United Kingdom) as well as Turkey and Russia.

b Includes 33 respondents from Japan, 13 from Australia, 7 from Brazil, 2 from New Zealand, 2 from India and 1 from Peru.

c Based on answering “yes” to the previous question.

### Candidate Trial Design Evaluation

**Figure 2:**
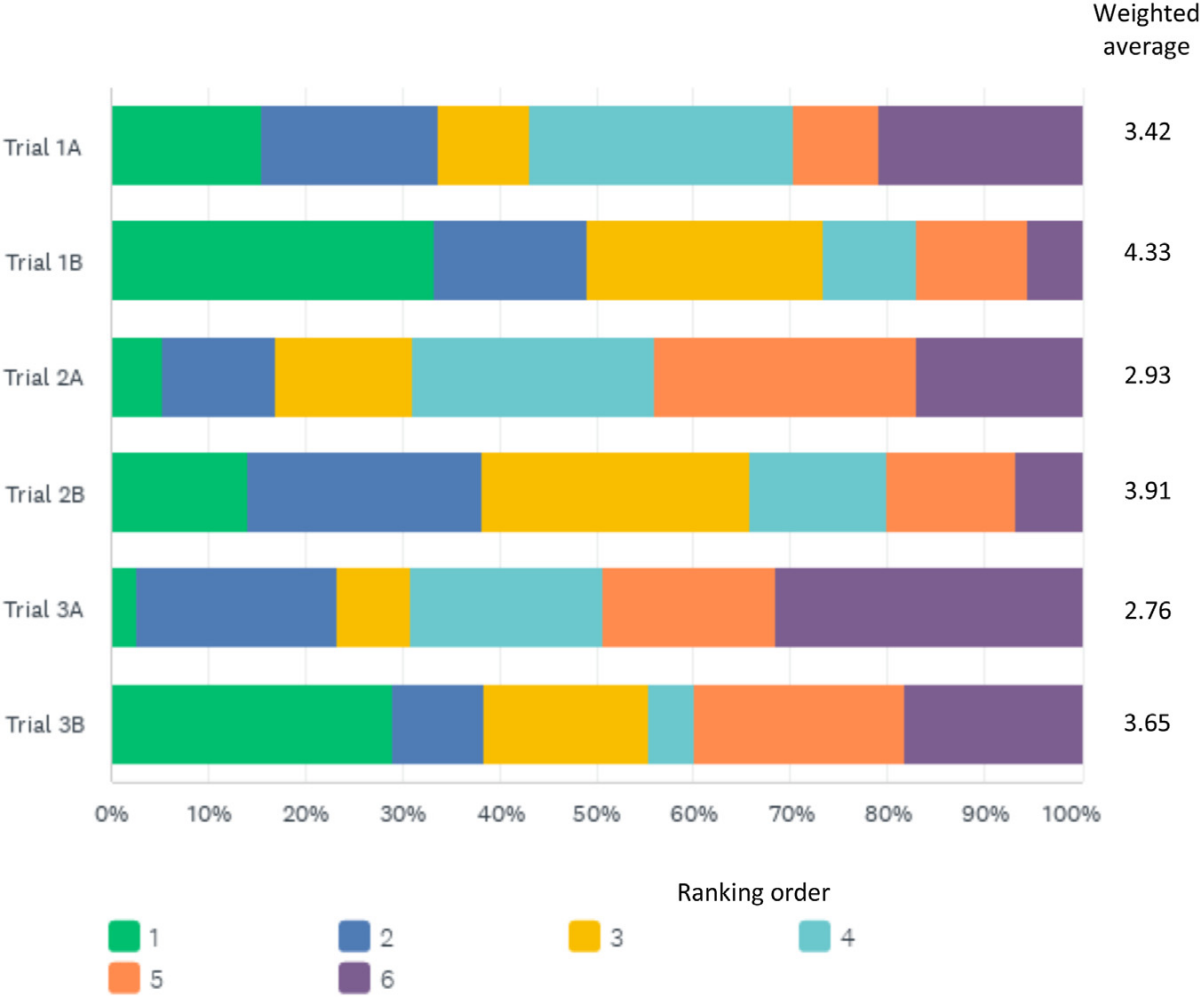
Overall candidate trial rankings. Weighted average score based on number of votes for a trial based on weight assigned from ranking of trial. Formula: [(X1 × W1)+(X2 × W2)+(X3 × W3)…] / T Xn = number of respondents for corresponding rank Wn = weight of corresponding rank T = total number of respondents Weights were applied in reverse: 6 ranks, so weighting up to 6, where rank 1 = weight of 6, rank 2 = weight of 5, rank 3 = weight of 4, etc.

Table 4 summarizes respondents’ assessment of each trial design. The 3 candidate trials were rated moderate-to-high for ability in assessing the safety and efficacy of minimal GC administration in severe AAV, with a slight preference for trial 1 (scores 7, 6, and 6 on 10-point Likert scale for trials 1, 2, and 3 respectively). Nephrologists had a higher rating compared to other specialties for trial 1 (8 vs 6, respectively) and trial 2 (7 vs 5, respectively), as did respondents from Ireland/United Kingdom for trial 1. The trials were rated moderate in terms of willingness to randomize patients into (scores of 6, 6, and 5 on 10-point Likert scale for trials 1, 2, and 3 respectively), with respondents from Ireland/United Kingdom having higher ratings for trials 1 and 2 compared to other geographic locations. For each trial design, the majority (62%-68%) of respondents felt that 4 weeks of GCs instead of 2 weeks would increase their willingness to randomize into the trial with only ∼5% stating it would make them less likely (Table 5). Overall trial ranking for preference to recruit patients showed that trial 1 scored highest and that the 4-week GC intervention arm options were preferred to the 2-week options, consistently across specialties and geographic locations (Fig 2; Table 6). The 4-week option seemed to have a greater impact in increasing trial ranking for rheumatologists, internists, and other specialties compared to nephrologists (Table 6).

**Table 4:** Evaluation of Candidate Trial Designs for Treatment of ANCA-associated Vasculitis

	Candidate Trial 1	Candidate Trial 2	Candidate Trial 3
	CYC-RTX combo + GC 2 wk vs GC PEXIVAS-reduced	CYC-RTX combo + GC 2 wk vs CYC or RTX + GC PEXIVAS-reduced	CYC and/or RTX + GC 2 wk vs GC PEXIVAS-reduced
Trial usefulness rating: “To what degree do you believe this trial design will answer the question of whether a minimal GC regimen for induction therapy of severe AAV, without avacopan, will be safe and effective?”; median (IQR)^a^			
Total	7 (5-8)	6 (4-8)	6 (4-8)
By subspecialty			
Nephrology	8 (5-8)	7 (5-8)	7 (5-8)
Rheumatology/GIM/Other	6 (4-8)	5 (4-8)	6 (4-8)
By geographic location			
Canada/United States	7 (5-8)	6 (5-8)	7 (5-8.25)
Ireland/United Kingdom	8 (5.5-8)	7 (4-8)	7 (3.75-8)
Europe^b^	7 (5.25-8)	7 (4-8)	7 (5-8)
Asia/Oceania/South America^c^	5 (3.25-7)	6 (4-7)	5 (3-8)
Willingness to randomize: “To what degree would you be willing to randomize participants with severe AAV into this trial design if the trial were sufficiently resourced?”; median (IQR)^a^			
Total	6 (3-8)	6 (3-8)	5 (3-8)
By subspecialty			
Nephrology	6 (4-8)	7 (4-8)	6 (3-8)
Rheumatology/GIM/Other	6 (3-8)	5 (3-7.5)	4 (3-7)
By geographic location			
Canada/United States	6 (3-8)	5.5 (3-8)	6 (4-7.25)
Ireland/United Kingdom	7 (5-8)	7 (3.75-8)	5.5 (2-7.25)
Europe^b^	6 (4-8)	5 (3-8)	6 (3-8)
Asia/Oceania/South America^c^	5 (3-7)	5 (4-7)	4 (2-7)

Abbreviations: AAV anti-neutrophil cytoplasmic antibody-associated vasculitis; ANCA, anti-neutrophil cytoplasmic antibody; CYC, cyclophosphamide; GC, glucocorticoid; GIM, general internal medicine; IQR, interquartile range; PEXIVAS, Plasma Exchange and Glucocorticoid Dosing in the Treatment of Anti-Neutrophil Cytoplasm Antibody Associated Vasculitis; RTX, rituximab.

a Based on scale from 1 to 10, where 1 represents being extremely unlikely and 10 being extremely likely.

b Includes all countries in Europe (except Ireland and the United Kingdom) as well as Turkey and Russia.

c Includes 33 respondents from Japan, 13 from Australia, 7 from Brazil, 2 from New Zealand, 2 from India, and 1 from Peru.

**Table 5:** Perception of 4-Week vs 2-Week Regimen for GC Minimization in ANCA-associated Vasculitis Induction Therapy

	Candidate Trial 1			Candidate Trial 2			Candidate Trial 3		
	CYC-RTX combo + GC 2 wk vs PEXIVAS-reduced GC			CYC-RTX combo + GC 2 wk vs CYC or RTX + PEXIVAS reduced GC			CYC and/or RTX + GC 2 wk vs PEXIVAS-reduced GC		
“How would a 4-week prednisone taper (ie, first wk 60 mg, second wk 30 mg, third week 20 mg, fourth week 10 mg, then stop) instead of 2-wk affect your willingness to randomize into this trial?”; n (%)									
	More likely	No change	Less likely	More likely	No change	Less likely	More likely	No change	Less likely
Total	104 (61.9%)	56 (33.3%)	8 (4.7%)	111 (65.3%)	50 (29.4%)	9 (5.3%)	117 (68.4%)	41 (23.9%)	13 (7.6%)
By subspecialty									
Nephrology	43 (58.9%)	28 (38.3%)	2 (2.7%)	43 (58.1%)	27 (36.5%)	4 (5.4%)	48 (64.9%)	21 (28.4%)	5 (6.8%)
Rheumatology/GIM/Other	61 (64.2%)	28 (29.5%)	6 (6.3%)	68 (70.8%)	23 (24.0%)	5 (5.2%)	69 (71.1%)	20 (20.6%)	8 (8.3%)
By geographic location									
Canada/United States	20 (60.6%)	12 (36.4%)	1 (3.0%)	21 (65.6%)	11 (34.4%)	0 (0%)	26 (81.3%)	6 (18.8%)	0 (0%)
Ireland/United Kingdom	16 (69.6%)	6 (26.1%)	1 (4.3%)	13 (56.5%)	8 (34.8%)	2 (8.7%)	12 (50.0%)	9 (37.5%)	3 (12.5%)
Europe^a^	43 (69.4%)	19 (30.7%)	0 (0%)	44 (80.0%)	17 (27.4%)	1 (1.6%)	47 (75.8%)	13 (21.0%)	2 (3.2%)
Asia/Oceania/South America^b^	25 (50.0%)	19 (38.0%)	6 (12.0%)	33 (62.3%)	14 (26.4%)	6 (11.3%)	32 (60.4%)	13 (24.5%)	8 (15.1%)

Abbreviations: ANCA, anti-neutrophil cytoplasmic antibody; CYC, cyclophosphamide; GC, glucocorticoid; GIM, general internal medicine; PEXIVAS, Plasma Exchange and Glucocorticoid Dosing in the Treatment of Anti-Neutrophil Cytoplasm Antibody Associated Vasculitis; RTX, rituximab.

a Includes all countries in Europe (except Ireland and the United Kingdom) as well as Turkey and Russia.

b Includes 33 respondents from Japan, 13 from Australia, 7 from Brazil, 2 from New Zealand, 2 from India, and 1 from Peru.

**Table 6:** Weighted Average Candidate Trial Overall Ranking, by Medical Specialty and by Geographic Location

	Candidate Trial 1		Candidate Trial 2		Candidate Trial 3	
	CYC-RTX combo + minimal GC vs PEXIVAS-reduced GC		CYC-RTX combo + minimal GC vs CYC or RTX + PEXIVAS-reduced GC		CYC and/or RTX + minimal GC vs PEXIVAS-reduced GC	
	2 wk GC	4 wk GC	2 wk GC	4 wk GC	2 wk GC	4 wk GC
Total	3.42	4.33	2.93	3.91	2.76	3.65
By subspecialty						
Nephrology	3.54	4.24	3.15	3.91	2.82	3.40
Rheumatology/GIM/Other	3.32	4.41	2.74	3.91	2.71	3.85
By geographic location						
Canada/United States	3.45	4.11	3.03	3.66	3.00	3.69
Ireland/United Kingdom	3.86	4.29	3.14	3.95	2.67	3.19
Europe^a^	3.48	4.39	2.74	3.74	2.83	3.81
Asia/Oceania/South America^b^	3.11	4.43	2.98	4.27	2.57	3.64

*Note*: Rankings were performed in response to the statement: “Please rank the following 6 candidate trial designs, where avacopan is NOT used, in order of preference for recruitment of patients with severe AAV (from most likely to want to recruit into, to least likely).”

Weighted average score was calculated with the number of votes for a trial based on weight assigned from ranking of trial.

Formula: [(X1 × W1)+(X2 × W2)+(X3 × W3)…] / T

Xn = number of respondents for corresponding rank

Wn = weight of corresponding rank

T = total number of respondents

Weights were applied in reverse: 6 ranks, so weighting up to 6, where rank 1 = weight of 6, rank 2 = weight of 5, rank 3 = weight of 4, etc.

Abbreviations: CYC, cyclophosphamide; GC, glucocorticoid; GIM, general internal medicine; PEXIVAS, Plasma Exchange and Glucocorticoid Dosing in the Treatment of Anti-Neutrophil Cytoplasm Antibody Associated Vasculitis; RTX rituximab.

a Includes all countries in Europe (except Ireland and the United Kingdom) as well as Turkey and Russia.

b Includes 33 respondents from Japan, 13 from Australia, 7 from Brazil, 2 from New Zealand, 2 from India, and 1 from Peru.

All free-text comments of the 3 candidate trial designs were reviewed. Of the 210 respondents, 49 provided comments for candidate trial 1, 54 for trial 2, and 58 for trial 3. Responses to all 3 trials highlighted that 2 weeks of GCs was not enough. The most common critiques of trials 1 and 2 were discomfort and unfamiliarity with the use of cyclophosphamide-rituximab combination or that such combination does not represent standard of care for induction therapy. The most common critique of trial 3 was heterogeneity from possible variations in cyclophosphamide and rituximab agent use.

## Discussion

Our survey provides insight into potential practice variations among clinicians and to what extent they are willing to minimize GC use for induction therapy in severe AAV. These results may help inform future trial development.

Our survey highlights potential differences in practice for managing AAV depending on medical specialty and geographic location. Nephrologists appear to be more open to minimal GC use compared to other specialties. This could reflect differences in the types of patients managed by different specialties and prior work on GC minimization. For example, rheumatologists or internists more commonly care for patients with prominent extra-renal manifestations, which may flare more frequently or be resistant to treatment, therefore influencing comfort in GC minimization. Also, comfort in GC minimization may be dictated by local practice, where the study by Pepper et al^5^ examining 1-2 weeks of GCs for induction therapy in AAV came from centers in Ireland and the United Kingdom. The higher rating for the candidate trials that used a combination of cyclophosphamide-rituximab in respondents from Ireland/United Kingdom could be due to the many observational studies examining this combination, which have come from those countries.^5^^,^^8^, ^9^, ^10^ However, centers in the United States have also published on this combination.^11^^,^^12^ Data from the RITUXVAS trial support the use of cyclophosphamide-rituximab combination,^13^ which is also recommended as a potential induction regimen in the 2022 Kidney Disease: Improving Global Outcomes (KDIGO) Glomerulonephritis guidelines.^14^ Despite these existing data, it was interesting to note the frequency of comments stating discomfort and unfamiliarity with this treatment option among our survey respondents. Nonetheless, combination cyclophosphamide-rituximab induction seemed the preferred trial design for examining GC minimization without avacopan in severe AAV. This combination regimen, potentially allowing for rapid reduction and withdrawal of GCs, would benefit from being studied in an RCT setting.

Our survey is informative of the perceptions on GC minimization among clinicians. First, there seems to be a limit to how low clinicians are willing to go in terms of GC dosing. Many respondents brought up concerns with only 2 weeks of GC use without avacopan for severe disease. The proposed 4-week taper option garnered more interest, and such a regimen would still equate to nearly a 60% reduction of the planned cumulative prednisone exposure compared to PEXIVAS-reduced dose. Such reduction in GCs could further reduce risks for infectious and GC-related complications, lower than that already seen in PEXIVAS (31% risk reduction in serious infections) and Low-dose Glucocorticoid Vasculitis Induction Study (absolute reductions of 13% for serious infections, 29% for all infections, and 21% for diabetes).^2^^,^^3^ Whether the addition of cyclophosphamide to rituximab increases immunosuppression burden and thereby masks some of the benefit from GC minimization to reduce infections remains unclear. However, use of low cumulative doses of cyclophosphamide as proposed in our survey should have a limited effect on infection risk, and the impact on reduction of GC-related adverse events should remain, although this has never been studied in an RCT setting. Second, our results underscore the impact of the PEXIVAS trial on establishing the standard of care for how GCs should be prescribed in severe AAV. Guidelines now recommend PEXIVAS-reduced dose taper for induction therapy when using GCs.^15^ The data from this survey confirm that clinicians with experience in vasculitis endorse these recommendations. Third, our survey highlights the growing role of avacopan in the treatment of AAV. The ADVOCATE trial was published less than 2 years before our survey, yet over 40% of respondents indicated they would not embark on a GC minimization trial without the use of avacopan. This further attests to the importance the clinical community places on reducing GC exposure in AAV.

Although avacopan represents a safe, effective means of doing so, it is difficult to predict how widespread its use will be in 5-10 years. The cost, availability, and uncertainty regarding its use in the most severe disease could limit widespread uptake. However, a reasonable assumption is that use will increase. The fact our survey was taken in the context of no avacopan use could explain why respondents only rated a moderate likelihood of wanting to randomize patients into any of our proposed GC minimization trials, despite these having been developed after careful consideration and consultation with experienced vasculitis researchers. Therefore, a GC minimization trial design may need to consider incorporating avacopan into the treatment arms to maintain clinician acceptability. An important point to consider though is that despite its growing popularity, cost is likely to limit availability for patients in many countries throughout the world. Demonstrating the safety and efficacy of minimal GCs for induction therapy in severe AAV, without the use of avacopan, remains relevant.

Though our results offer a better understanding of perceptions on GC minimization within the vasculitis community, with respondents from many countries and multiple continents, there are limitations to consider. Survey respondents were contacted through multiple sources, such that an individual could have received multiple invitations to participate. We could not calculate response rates from the various societies through which the survey was disseminated. Although we specified the survey was to be completed only by clinicians who manage patients with AAV, we cannot say whether the respondents differ systematically from non-respondents and therefore cannot say how representative it is of the average clinician. However, the responses align with expert opinions expressed in public forums and current evidence suggesting a degree of consistency between the relatively broad group of respondents and the expert community. There were very few respondents from developing countries. Clinicians in such countries may have varying availability of standard induction agents such as rituximab, not to mention avacopan. Therefore, the importance of avacopan and possible interest in rituximab-cyclophosphamide combination therapy may not be generalizable to the vasculitis community at large.

Our survey of vasculitis clinicians on the optimal trial design for studying GC minimization in severe AAV without the use of avacopan revealed that cyclophosphamide-rituximab as combination induction agents may be the optimal way of studying this. Studying such a treatment regimen in an RCT would be useful to examine potential benefits in terms of GC minimization and infection risks. We also wonder if certain differences in practice based on the types of patients managed and local practice patterns may influence a clinician’s degree of comfort with this combination regimen and with the degree of GC minimization one would be willing to tolerate. A 4-week GC taper seems to be a reasonable compromise between GC minimization and clinician acceptability. The place of avacopan in a GC minimization trial must be considered despite its limited availability for many patients in many countries. Its uptake seems to be increasing quickly and could be even greater within the next 3-5 years.

## Supplementary Materials

Supplementary File (PDF) Item S1-S2.

## References

[1] Robson J.C., Dawson J., Cronholm P.F. (2018). Patient perceptions of glucocorticoids in anti-neutrophil cytoplasmic antibody-associated vasculitis. Rheumatol Int.

[2] Furuta S., Nakagomi D., Kobayashi Y. (2021). Effect of reduced-dose vs high-dose glucocorticoids added to rituximab on remission induction in ANCA-associated vasculitis: a randomized clinical trial. JAMA.

[3] Walsh M., Merkel P.A., Peh C.A. (2020). Plasma exchange and glucocorticoids in severe ANCA-associated vasculitis. N Engl J Med.

[4] Jayne D.R.W., Merkel P.A., Schall T.J., Bekker P., ADVOCATE Study Group (2021). Avacopan for the treatment of ANCA-associated vasculitis. N Engl J Med.

[5] Pepper R.J., McAdoo S.P., Moran S.M. (2019). A novel glucocorticoid-free maintenance regimen for anti-neutrophil cytoplasm antibody-associated vasculitis. Rheumatol.

[6] Brimble K.S., Mehrotra R., Tonelli M. (2013). Estimated GFR reporting influences recommendations for dialysis initiation. J Am Soc Nephrol.

[7] Burns K.E.A., Duffett M., Kho M.E. (2008). A guide for the design and conduct of self-administered surveys of clinicians. CMAJ.

[8] Mcadoo S.P., Medjeral-Thomas N., Gopaluni S. (2019). Long-term follow-up of a combined rituximab and cyclophosphamide regimen in renal anti-neutrophil cytoplasm antibody-associated vasculitis. Nephrol Dial Transplant.

[9] Gulati K., Edwards H., Prendecki M. (2021). Combination treatment with rituximab, low-dose cyclophosphamide and plasma exchange for severe antineutrophil cytoplasmic antibody-associated vasculitis. Kidney Int.

[10] Mansfield N., Hamour S., Habib A.M. (2011). Prolonged disease-free remission following rituximab and low-dose cyclophosphamide therapy for renal ANCA-associated vasculitis. Nephrol Dial Transplant.

[11] McGregor J.G., Hogan S.L., Kotzen E.S. (2015). Rituximab as an immunosuppressant in antineutrophil cytoplasmic antibody-associated vasculitis. Nephrol Dial Transplant.

[12] Cortazar F.B., Muhsin S.A., Pendergraft W.F. (2018). Combination therapy with rituximab and cyclophosphamide for remission induction in ANCA vasculitis. Kidney Int Rep.

[13] Jones R.B., Cohen Tervaert J.W., Hauser T. (2010). Rituximab versus cyclophosphamide in ANCA-associated renal vasculitis. N Engl J Med.

[14] Kidney Disease: Improving Global Outcomes (KDIGO) Glomerular Diseases Work Group (2021). KDIGO 2021 Clinical Practice Guideline for the Management of Glomerular Diseases. Kidney Int.

[15] Casal Moura M., Gauckler P., Anders H.J. (2023). Management of antineutrophil cytoplasmic antibody-associated vasculitis with glomerulonephritis as proposed by the ACR 2021, EULAR 2022 and KDIGO 2021 guidelines/recommendations. Nephrol Dial Transplant.

