# Supplementary material for: Glucocorticoid Minimization in Anti-Neutrophil Cytoplasmic Antibody-Associated Vasculitis: An International Survey of Clinicians
Source: Kidney Med. 2024 Jun 14;6(8):100858. doi: 10.1016/j.xkme.2024.100858 (PMC11315210; doi:10.1016/j.xkme.2024.100858)
Supplement: Supplementary File (PDF) — Item S1-S2. [file mmc1.pdf]

Appendix

Item S1. First iteration survey clinical sensibility testing results

Survey clinical sensibility assessment

Question 1 (Face validity)
To what extent were the survey questions directed at the issue of developing an optimal trial design examining minimal GC use for induction therapy in severe ANCA vasculitis?
1. Trivial extent
2. Small extent
3. Fair extent
4. Moderate extent
5. Large extent

Trivial extent	0.00%	0
Small extent	0.00%	0
Fair extent	0.00%	0
Moderate extent	22.22%	2
Large extent	77.78%	7

Question 2 (Content validity)
Are there important aspects about developing an optimal trial design examining minimal GC use for induction therapy in severe ANCA vasculitis which were not addressed in the survey?
1. Crucial gaps
2. Important gaps
3. Minor gaps
4. Minimal gaps
5. Insignificant gaps

Crucial gaps	0.00%	0
Important gaps	33.33%	3
Minor gaps	33.33%	3
Minimal gaps	22.22%	2
Insignificant gaps	11.11%	1

Question 3 (Clarity)
To what extent were the response options simple and easily understood?
a) Trivial extent
b) Small extent
c) Fair extent
d) Moderate extent

e) Large extent

Trivial extent	0.00%	0
Small extent	0.00%	0
Fair extent	22.22%	2
Moderate extent	22.22%	2
Large extent	55.56%	5

Question 4 (Utility)
To what extent were the questions likely to elicit candid information from the respondents?
a) Trivial extent
b) Small extent
c) Fair extent
d) Moderate extent
e) Large extent

Trivial extent	0.00%	0
Small extent	0.00%	0
Fair extent	0.00%	0
Moderate extent	22.22%	2
Large extent	77.78%	7

Question 5 (Redundancy)
How many items were inappropriate or redundant?
a) Very many
b) Many
c) Some
d) A few
e) Not many

Very many	0.00%	0
Many	0.00%	0
Some	22.22%	2
A few	11.11%	1
Not many	66.67%	6

Item S2. Final survey

Steroid minimization in **A**NCA-associated vasculitis **F**or safe **E**ffective **T**reatment – the SAFE-T trial: A survey of vasculitis clinicians

Purpose

Efforts have been made to reduce the cumulative dose of glucocorticoids (GC) during induction treatment of ANCA-associated vasculitis (AAV). The PEXIVAS and LOVAS trials demonstrated that marked reduction of GC dose, compared to what was previously the standard of care, is effective in inducing remission of moderate to severe AAV. Newer agents, such as avacopan, may allow further reduction in GC exposure for some patients with AAV. Non-controlled studies have suggested that minimal GC (as little as 1-2 weeks duration) combined with both cyclophosphamide and rituximab may also effectively induce remission of AAV. However, there are no randomized trials evaluating such a minimal GC regimen. The feasibility of studying such a strategy in a randomized trial and what the treatment and comparators would optimally be remains uncertain.

The purpose of this survey is to inform the development of a clinical trial examining a minimal GC regimen for induction therapy in severe AAV, with the aims of achieving effective induction of remission while significantly reducing glucocorticoid-associated toxicity. The study population will include individuals with severe, organ-threatening manifestations of AAV (rapidly progressive glomerulonephritis, pulmonary hemorrhage, central nervous system, ocular, cardiac or gastrointestinal involvement, or mononeuritis multiplex).

This survey will allow us to explore variations in practice and to better understand concerns among vasculitis clinicians regarding the use of GC as well as the types and doses of adjunct induction agents used in AAV. It takes approximately 15 minutes to complete and consists of general questions followed by specific questions pertaining to potential trial designs. We intend to use the results of the survey to design a clinical trial which will be acceptable to clinicians in terms of patient recruitment and which will be of sufficient interest within the vasculitis community. Your participation is voluntary and all responses are confidential.

Respondent demographics

1. In what country do you work?
(Survey monkey list of countries)

2. What is your primary location of clinical practice?
a) Academic
b) Non-academic

3. In what year did you or will you begin independent practice (no longer training)?
(number only answer)

4. What is your primary medical specialty?
a) Rheumatology
b) Nephrology
c) General Internal Medicine
d) Other

5. Please estimate the number of times you started induction therapy for the treatment of AAV in the last 12 months
(number only answer)

6. Please estimate the number of patients with AAV you would see on follow-up in an outpatient setting over 12 months in your clinical practice
(number only answer)

7. How many clinical trials of AAV have you been involved in as a site investigator for patient recruitment?
(number only answer)

General trial design questions

1. Please state your agreement with the following statement: a reduced-dose GC taper similar to what was used in PEXIVAS is the **current standard-of-care** for GC use in patients with severe AAV
a) Completely agree
b) Somewhat agree
c) Unsure
d) Somewhat disagree
e) Completely disagree

2. We acknowledge that some centres may experience difficulties in accessing certain induction therapies due to funding restrictions or logistical constraints such as access to infusion clinics. Please state your agreement with the following statements:

2.i. If IV cyclophosphamide had to be used in the intervention and/or control arms, but **were not provided by the trial**, I would not be able to recruit patients into a GC minimisation trial
a) Completely agree
b) Somewhat agree
c) Unsure
d) Somewhat disagree
e) Completely disagree

2.ii. If rituximab had to be used in the intervention and/or control arms, but **were not provided by the trial**, I would not be able to recruit patients into a GC minimisation trial
a) Completely agree
b) Somewhat agree
c) Unsure
d) Somewhat disagree
e) Completely disagree

3. Do you feel that the use of avacopan **must** be permitted in any AAV GC minimisation trial design for you to want to recruit a patient into such a trial?
a) Yes
b) No

4. Do you agree with the following statement? **Two weeks** of GC for induction therapy in severe AAV, without using avacopan and irrespective of the amount of cyclophosphamide and/or rituximab used, is **too little GC** for me to want to recruit patients into a trial.
a) Yes
b) No

4.i. (if option "Yes" is selected) What would be the minimal number of weeks of GC that would make a trial acceptable to you?
(number only answer)

Specific candidate trial questions

In the following section, you will be presented with a brief table and pictogram description for 3 potential trial designs comparing a minimal GC regimen to the PEXIVAS reduced dose regimen. The trial designs differ in terms of the adjunct immunosuppressive agents (CYC and/or RTX) used in the study arms; please note that avacopan is **NOT** used in any of these potential trials. After each description, you will have 4 questions about the appeal of the design to you.

Candidate trial 1

CYC-RTX combo + GC 2 weeks vs GC PEXIVAS low

	**Intervention arm**	**Control arm**
**Induction therapy**	2 doses, 2 weeks apart each of IV CYC and RTX	
Methylprednisolone (optional): 0-3 daily pulses, dose at investigator choice		
**Glucocorticoid (blinded placebo control)**	W1 60mg daily<br>W2 30mg daily	PEXIVAS reduced-dose taper

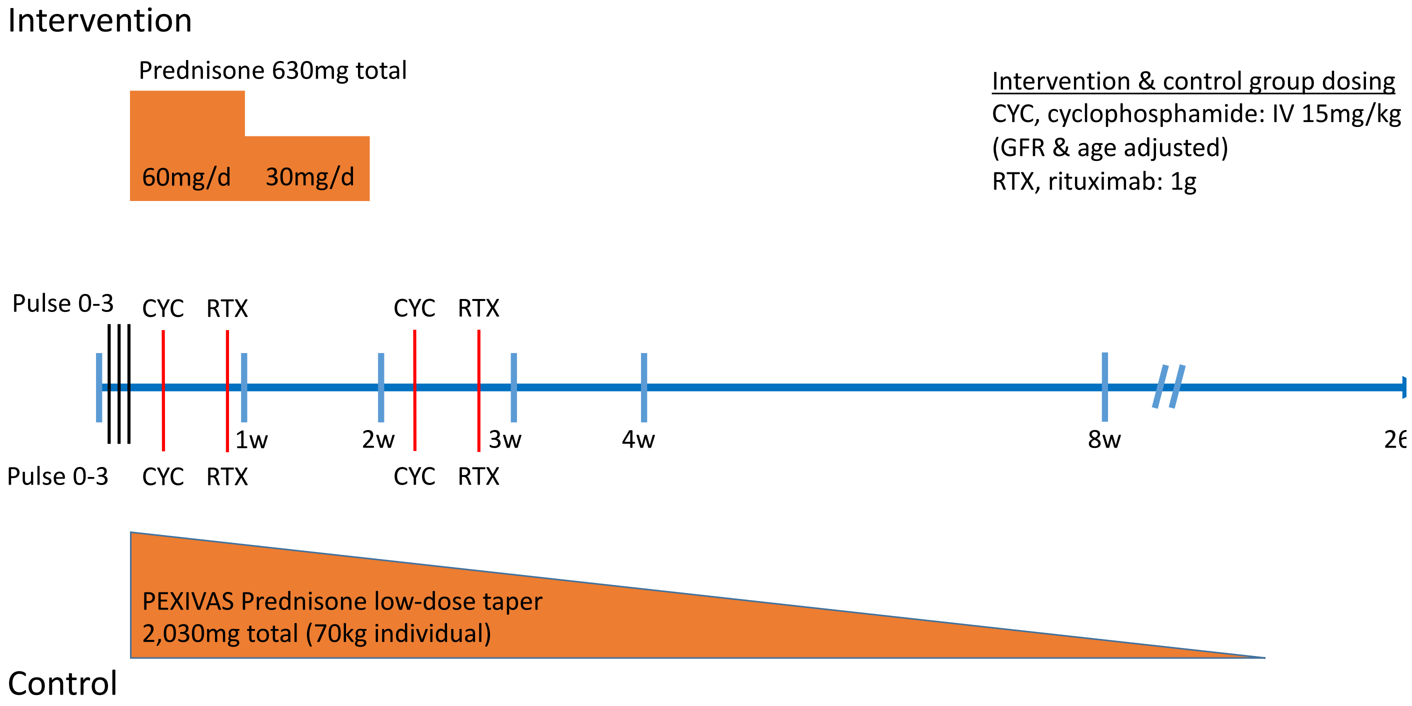

1. To what degree do you believe the above design will answer the question of whether a minimal GC regimen for induction therapy of severe AAV, without avacopan, will be safe and effective.
(bar scale 1-10, 1 being extremely unlikely, 10 being extremely likely)

2. To what degree would you be willing to randomize participants with severe AAV into the above design if the trial were sufficiently resourced
(bar scale 1-10, 1 being extremely unlikely, 10 being extremely likely)

3. Consider the above trial design but instead with a 4-week taper of prednisone: 1st week 60mg, 2nd week 30mg, 3rd week 20mg, 4th week 10mg, then stop. How would the 4-week instead of 2-week duration of prednisone affect your willingness to randomize participants with severe AAV into this trial?
a) More likely
b) No change
c) Less likely

4. Please leave any comments you may have about the above trial design (optional response)
(free text)

Candidate trial 2

CYC-RTX combo + GC 2 weeks vs CYC or RTX + GC PEXIVAS low

	**Intervention arm**	**Control arm**
**Induction therapy**	2 doses, 2 weeks apart each of IV CYC and RTX	Pre-specified PO CYC, IV CYC or RTX (investigator choice)
Methylprednisolone (optional): 0-3 daily pulses, dose at investigator choice		
**Glucocorticoid (open-label)**	W1 60mg daily<br>W2 30mg daily	PEXIVAS reduced-dose taper

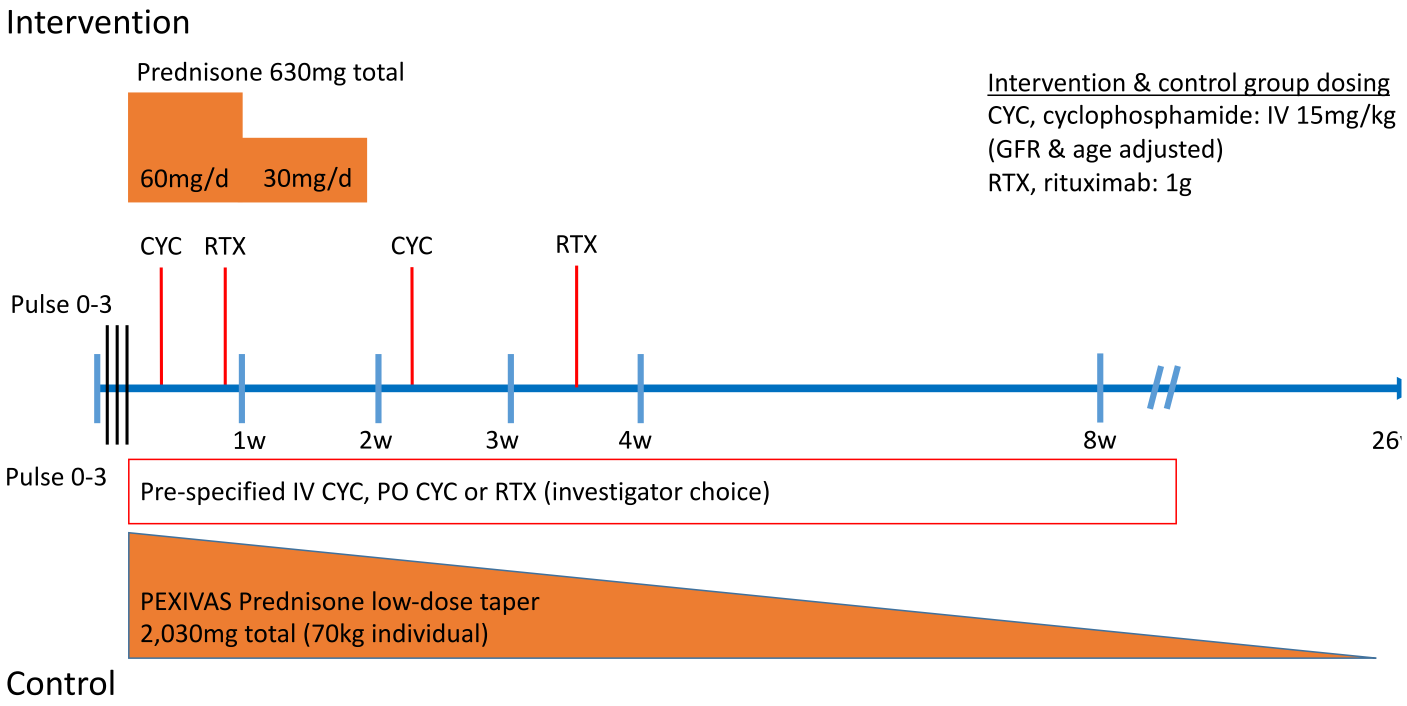

1. To what degree do you believe the above design will answer the question of whether a minimal GC regimen for induction therapy of severe AAV, without avacopan, will be safe and effective.
(bar scale 1-10, 1 being extremely unlikely, 10 being extremely likely)

2. To what degree would you be willing to randomize participants with severe AAV into the above design if the trial were sufficiently resourced

(bar scale 1-10, 1 being extremely unlikely, 10 being extremely likely)

3. Consider the above trial design but instead with a 4-week taper of prednisone: 1st week 60mg, 2nd week 30mg, 3rd week 20mg, 4th week 10mg, then stop. How would the 4-week instead of 2-week duration of prednisone affect your willingness to randomize participants with severe AAV into this trial?
a) More likely
b) No change
c) Less likely

4. Please leave any comments you have about the above trial design (optional response)
(free text)

Candidate trial 3

CYC and/or RTX + GC 2 weeks or GC PEXIVAS low

	**Intervention arm**	**Control arm**
**Induction therapy**	PO CYC, IV CYC, RTX or CYC-RTX combo at investigator choice	
Methylprednisolone (optional): 0-3 daily pulses, dose at investigator choice		
**Glucocorticoid (blinded placebo control)**	W1 60mg daily<br>W2 30mg daily	PEXIVAS reduced-dose taper

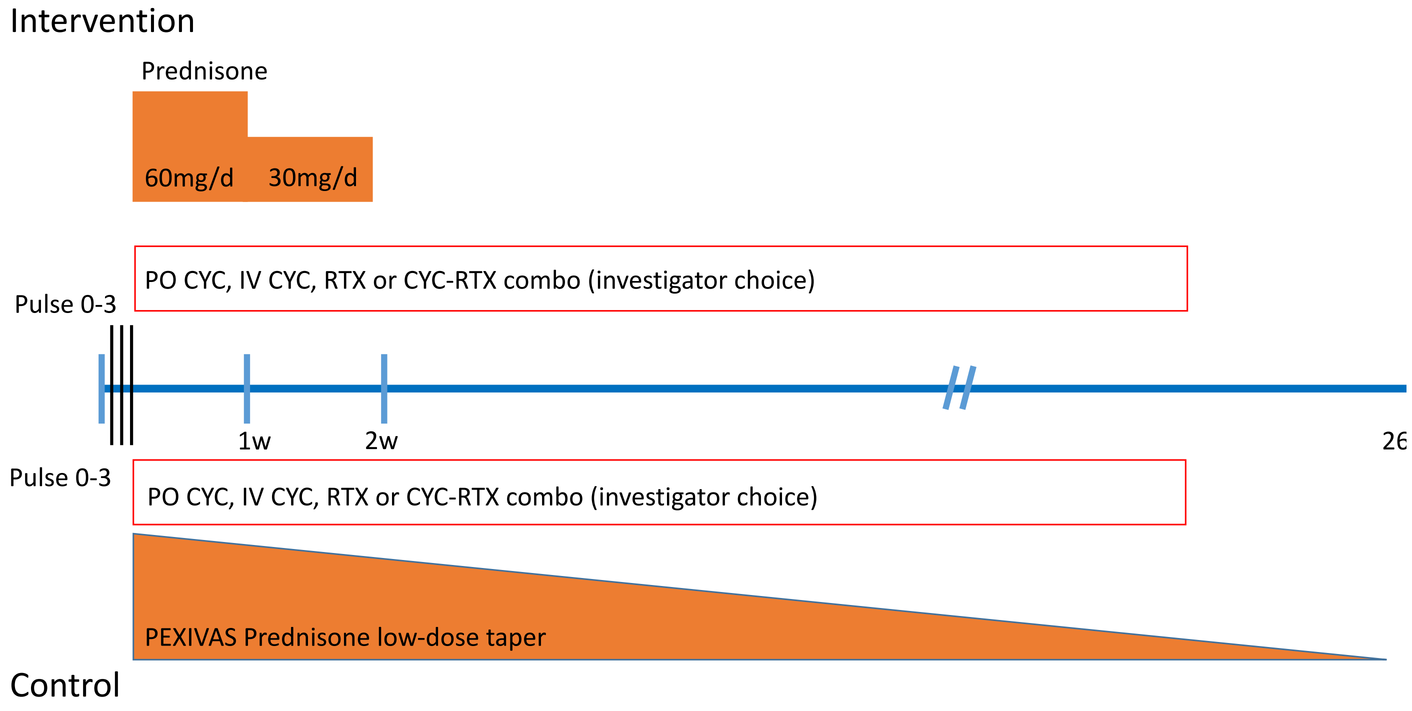

1. To what degree do you believe the above design will answer the question of whether a minimal GC regimen for induction therapy of severe AAV, without avacopan, will be safe and effective.
(bar scale 1-10, 1 being extremely unlikely, 10 being extremely likely)

2. To what degree would you be willing to randomize participants with severe AAV into the above design if the trial were sufficiently resourced
(bar scale 1-10, 1 being extremely unlikely, 10 being extremely likely)

3. Consider the above trial design but instead with a 4-week taper of prednisone: 1st week 60mg, 2nd week 30mg, 3rd week 20mg, 4th week 10mg, then stop. How would the 4-week instead of 2-week duration of prednisone affect your willingness to randomize participants with severe AAV into this trial?
a) More likely
b) No change
c) Less likely

4. Please leave any comments you have about the above trial design (optional response)
(free text)

Candidate trial ranking

1. You were just presented with 3 different trial designs, and asked about willingness to recruit based on 2 different durations of GC (2 weeks and 4 weeks). Please rank the following 6 candidate trial designs, where avacopan is NOT used, in order of preference for recruitment of patients with severe AAV (from most likely to want to recruit into, to least likely)

a) Trial 1A
b) Trial 1B
c) Trial 2A
d) Trial 2B
e) Trial 3A
f) Trial 3B

**Trial 1A**		
	**Intervention arm**	**Control arm**
**Induction therapy**	2 doses, 2 weeks apart each of IV CYC and RTX	
**Glucocorticoid**	W1 60mg daily<br>W2 30mg daily	PEXIVAS reduced-dose taper

**Trial 1B**		
	**Intervention arm**	**Control arm**
**Induction therapy**	2 doses, 2 weeks apart each of IV CYC and RTX	
**Glucocorticoid**	W1 60mg daily<br>W2 30mg daily<br>W3 20mg daily<br>W4 10mg daily	PEXIVAS reduced-dose taper

**Trial 2A**		
	**Intervention arm**	**Control arm**
**Induction therapy**	2 doses, 2 weeks apart each of IV CYC and RTX	Pre-specified PO CYC, IV CYC or RTX (investigator choice)
**Glucocorticoid**	W1 60mg daily<br>W2 30mg daily	PEXIVAS reduced-dose taper

**Trial 2B**		
	**Intervention arm**	**Control arm**
**Induction therapy**	2 doses, 2 weeks apart each of IV CYC and RTX	Pre-specified PO CYC, IV CYC or RTX (investigator choice)
**Glucocorticoid**	W1 60mg daily<br>W2 30mg daily<br>W3 20mg daily<br>W4 10mg daily	PEXIVAS reduced-dose taper

**Trial 3A**		
	**Intervention arm**	**Control arm**
**Induction therapy**	PO CYC, IV CYC, RTX or CYC-RTX combo at investigator choice	

**Glucocorticoid**	W1 60mg daily<br>W2 30mg daily	PEXIVAS reduced-dose taper

**Trial 3B**		
	**Intervention arm**	**Control arm**
**Induction therapy**	PO CYC, IV CYC, RTX or CYC-RTX combo at investigator choice	
**Glucocorticoid**	W1 60mg daily<br>W2 30mg daily<br>W3 20mg daily<br>W4 10mg daily	PEXIVAS reduced-dose taper
